# Elobixibat alleviates chronic constipation in hemodialysis patients: a questionnaire-based study

**DOI:** 10.1186/s12876-020-1179-6

**Published:** 2020-01-31

**Authors:** Daigo Kamei, Yuiko Kamei, Masashi Nagano, Michio Mineshima, Kosaku Nitta, Ken Tsuchiya

**Affiliations:** 1 Department of Nephrology, Tokyo Women’s Medical University, 8-1 Kawada-cho, Shinjuku-ku, Tokyo, 162-8666 Japan; 2 Department of Blood Purification, Kidney Center, Tokyo Women’s Medical University, 8-1 Kawada-cho, Shinjuku-ku, Tokyo, 162-8666 Japan; 3 Department of Clinical Engineering, Tokyo Women’s Medical University, 8-1 Kawada-cho, Shinjuku-ku, Tokyo, 162-8666 Japan; 4 Nerima Sakuradai Clinic, 4-11-9 Toyotamakita, Nerima-ku, Tokyo, 176-0012 Japan

**Keywords:** Hemodialysis patients, Constipation, Elobixibat, Bristol stool form scale, Patient assessment of constipation-quality of life questionnaire

## Abstract

**Background:**

Hemodialysis patients are prone to constipation, which can adversely affect their quality of life (QOL). Elobixibat, a highly selective inhibitor of the ileal bile acid transporter, can increase the bile acid level in the colon and, subsequently, enhance colonic motility and secretion. In hemodialysis patients with chronic constipation, it may have a novel action mechanism. However, the effect of elobixibat on such patients’ QOL had not been reported. This study aimed to evaluate the effect of elobixibat on the QOL of hemodialysis patients with chronic constipation.

**Methods:**

This was a multicenter, observational study that used the Japanese version of the Patient Assessment of Constipation-Quality of Life (PAC-QOL) questionnaire on 27 patients (18 men and nine women, age range 47–90 years), who satisfied the Rome 3 diagnostic criteria for functional constipation and were already taking other drugs for constipation. These patients were administered elobixibat 10 mg/day and were asked to respond to the PAC-QOL questionnaire at baseline and after 4 weeks. Bayesian statistics were used to confirm our results.

**Results:**

The number of spontaneous bowel movements per week increased significantly from 2.6 ± 1.2 to 4.1 ± 2.1 (*p* < 0.001), and the Bristol Stool Form Scale score significantly improved from 1.9 ± 0.8 to 3.6 ± 0.7 (*p* < 0.001). The Cronbach’s alpha was 0.95, and the Guttman split-half reliability coefficient was 0.90. There were significant decreases in the physical discomfort scores from 1.94 ± 0.79 to 0.97 ± 0.72 (*p* < 0.001); psychosocial discomfort from 1.16 ± 0.93 to 0.63 ± 0.58 (*p* < 0.001); worries/ concerns from 1.84 ± 0.73 to 1.27 ± 0.59 (*p* < 0.001), and satisfaction from 2.79 ± 0.61 to 1.98 ± 0.77 (*p* < 0.001). The total PAC-QOL score significantly decreased from 1.83 ± 0.79 to 1.17 ± 0.56 (*p* < 0.001). Bayesian statistics confirmed the results’ significance.

**Conclusions:**

Elobixibat reduced the PAC-QOL scores for hemodialysis patients with chronic constipation and improved the patients’ QOL. It may serve as a new option for treating constipation in hemodialysis patients.

## Background

Chronic constipation is a common condition that may suggest digestive tract lesions, such as colon cancer, or systemic diseases, such as thyroid disease. As the elderly population increases, so does the number of patients with chronic constipation. Chronic constipation greatly impairs a patient’s quality of life (QOL), and improving it can address both physical and mental QOL [1]. The prevalence of chronic constipation in dialysis patients based on evaluations using the Gastrointestinal Symptom Rating Scale was reported to be 36.3–66.7% [2]. In hemodialysis patients, constipation causes include water restriction, water removal by dialysis, inadequate intake of dietary fiber due to potassium restriction and the associated changes in intestinal microflora, lack of exercise, decreased intestinal tract motility due to aging and muscle weakness, diabetic autonomic nervous system disorder, intake of potassium inhibitors and phosphorus adsorbents, and tolerance due to abuse of irritant laxatives.

Elobixibat, a novel local-acting and highly selective inhibitor of the ileal bile acid transporter that is expressed in the terminal ileum, had been used to treat chronic constipation and was shown to increase bile acid levels in the colon, subsequently enhancing colonic motility and secretion [3–6]. However, there had been no reports on the efficacy of elobixibat in hemodialysis patients with chronic constipation. This aim of this study was to assess the efficacy of elobixibat on the QOL and its effect on the Bristol Stool Form Scale (BSFS) score and the number of spontaneous bowel movements (SBMs) per week of dialysis patients with chronic constipation.

## Methods

### Subjects

We enrolled patients who answered the questionnaire before and after elobixibat intake and who had taken elobixibat in addition to other laxatives to relieve the symptoms of chronic constipation. This retrospective observational study was conducted by collecting data from May 2018 to May 2019. We investigated 27 patients who were on maintenance dialysis three times per week. Three patients underwent dialysis at the Tokyo Women’s Medical University and 24 patients did at the Nerima Sakuradai Clinic. All patients satisfied the Rome 3 diagnostic criteria for functional constipation and were treated in accordance with the clinical guidelines of the Japanese Society for Dialysis Therapy [7–11]. Written informed consent was obtained from each study subject. The study’s protocol was approved by Tokyo Women’s Medical University’s ethics committee and was conducted in accordance with the 2000 revised Helsinki Declaration of 1975.

### Data collection

Age, sex, weight, etiology of end-stage renal disease, dialysis duration, comorbidities, laboratory data, the Japanese version of the Patient Assessment of Constipation-Quality of Life (PAC-QOL) questionnaire [12, 13], self-reported BSFS score [14], and the number of SBMs per week were obtained from the patients’ medical records. The conventional kinetic measure for urea, known as the Kt/V (single pool), was used to estimate the dialysis dose. The values of thyroid stimulating hormone, free triiodothyronine, free thyroxine, and beta-2 microglobulin were based on the latest blood collection.

### Statistical analysis

Data were presented as mean ± standard deviation and as medians and interquartile range. The PAC-QOL score, BSFS score, and the number of SBMs per week before and after 4 weeks of elobixibat intake were compared using the Wilcoxon’s rank-sum test. Kendall rank correlation coefficient was used to examine bivariate associations. According to the recommendations the of American Statistical Association on *P* values [15, 16], we used the SPSS Bayesian methods to confirm the stability and robustness of our results. A value of *p* < 0.005 was considered statistically significant. All statistical analyses were performed with SPSS version 25.0 (IBM Corp.; Armonk, NY, USA).

## Results

Twenty-seven patients were enrolled in this study. Tables 1 and 2 show the characteristics of enrolled patients. There were 18 men and nine women. The mean age was 70 years, and the mean dialysis duration was 8 years. There were no patients with diseases that caused colonic stenosis, such as colon cancer and Crohn’s disease by abdominal echography, abdominal CT, or colonoscopy. There were no patients with biliary atresia and Parkinson’s disease. The types of the other laxatives taken were one in 13 patients, two in 10 patients, and three in 4 patients. There were no patients with liver dysfunction. The patients did not show any liver dysfunction, psychoneurotic or circulatory illness, allergy, blood cell abnormalities, or increased Creatine phosphokinase after taking Elobixibat.

**Table 1:** The characteristics of enrolled patients

Variable	Mean ± SD and Median [IQR]
Number of enrolled patients	27
Sex (Male/Female)	18/9
Etiology of end-stage renal disease	Diabetic nephropathy, 12 patients
	Nephrosclerosis, 6 patients
	CGN, 6 patients
	ADPKD, 2 patients
	Graft loss, 1 patient
Age (year)	70 ± 13, 75 [57, 80]
Dialysis duration (year)	8 ± 9, 6 [1,11]
Dry weight (kg)	58.1 ± 13.3, 58.1 [53.7, 61.5]
Single pool Kt/V	1.08 ± 0.25, 1.11 [0.86, 1.30]
Concomitant laxative	1 type drug: 13 patients
	2 types drugs: 10 patients
	3 types drugs: 4 patients
Type of concomitant laxative	Sennosides A and B, 26 patients
	Sodium picosulfate hydrate, 5 patients
	Glycerine enema, 1 patient
	Lactulose, 2 patients
	Sodium dihydrogen phosphate/sodium hydrogen carbonate, 2 patients
	Lubiprostone, 5 patients
	Lactomin, 3 patients
	Daikenchuto, 1 patient
Phosphate binder	0 drug, 3 patients
	1 type of drug, 15 patients
	2 types of drug, 9 patients
Type of phosphate binder	Lantern carbonate, 6 patients
	Calcium carbonate, 19 patients
	Ferric citrate, 6 patients
	Sucroferric oxyhydroxide, 2 patients

*CGN* chronic glomerulonephritis, *ADPKD* autosomal dominant polycystic kidney disease

**Table 2:** The laboratory data of enrolled patients

Variable	Mean ± SD, median [IQR]
Hemoglobin (g/dL)	10.7 ± 1.2, 10.7 [9.9, 11.7]
Total protein (g/dL)	6.3 ± 0.5, 6.4 [6.0, 6.6]
Albumin (g/dL)	3.4 ± 0.4, 3.4 [3.2, 3.6]
Blood urea nitrogen (mg/dL)	56.0 ± 13.2, 56.4 [45.1, 68.9]
Creatinine (mg/dL)	9.3 ± 3.0, 9.8 [6.9, 10.8]
Uremic acid (mg/dL)	7.1 ± 1.2, 7.3 [6.3, 7.9]
Aspartate aminotransferase (U/L)	14 ± 7, 12 [10, 14]
Alanine aminotransferase (U/L)	10 ± 6, 8 [7, 13]
Alkaline phosphatase (U/L)	251 ± 102, 217 [179, 291]
Lactate dehydrogenase (U/L)	200 ± 55, 191 [169, 208]
γ-glutamyltransferase (U/L)	24 ± 21, 17 [13, 25]
Total bilirubin (mg/dL)	0.3 ± 0.1, 0.2 [0.2, 0.3]
Cholinesterase (U/L)	195 ± 60, 182 [172, 219]
Creatine phosphokinase (U/L)	93 ± 75, 67 [45, 113]
Iron (μg/dL)	54 ± 25, 45 [35, 76]
Total iron binding capacity (μg/dL)	249 ± 46, 248 [215, 277]
Ferritin (ng/mL)	140 ± 148, 82 [32, 211]
Thyroid stimulating hormone (μU/mL)	2.13 ± 1.71, 1.55 [1.01, 2.59]
Free triiodothyronine (pg/mL)	1.97 ± 0.38, 2.04 [1.75, 2.29]
Free thyroxine (ng/dL)	1.00 ± 0.19, 1.01 [0.90, 1.09]
Intact parathyroid hormone (pg/mL)	155 ± 138, 110 [70, 203]
Beta 2 microglobulin (mg/L)	28.4 ± 9.1, 26.6 [23.1, 31.2]

**Fig. 1:**
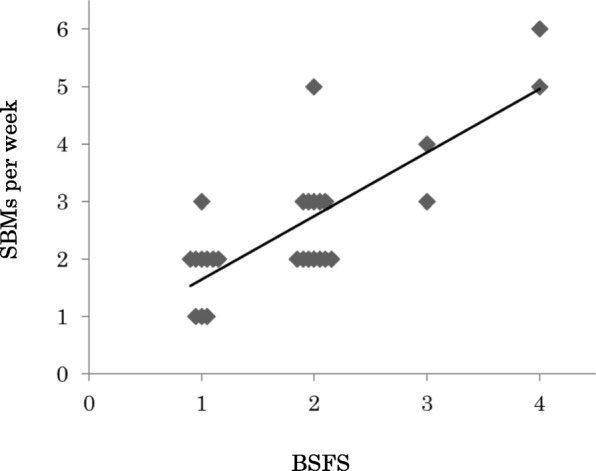
BSFS and SBMs in baseline. Kendall rank correlation coefficient was 0.650 (*P* < 0.001). SBMs, spontaneous bowel movements; BSFS, Bristol Stool Form Scale

Figure 1 shows the relationship between BSFS and SBMs at the baseline. BSFS and SBMs were significantly related (Kendall rank correlation coefficient tau = 0.650, *p* < 0.001). Bayesian statistics confirmed the significance of the result.

The internal consistency test result (Cronbach’s alpha) of 0.956 and the reliability coefficient of 0.899, obtained by Guttman’s split-half method, confirmed the PAC-QOL questionnaire’s reliability. Table 3 shows the PAC-QOL scores, SBMs, and BSFS score at baseline and after 4 weeks of elobixibat 10 mg/day intake. There were significant decreases in the scores for physical discomfort from 1.94 ± 0.79 to 0.97 ± 0.72 (*p* < 0.001); psychosocial discomfort from 1.16 ± 0.93 to 0.63 ± 0.58 (*p* < 0.001); worries/ concerns from 1.84 ± 0.73 to 1.27 ± 0.59 (*p* < 0.001); and satisfaction scores from 2.79 ± 0.61 to 1.98 ± 0.77 (*p* < 0.001). The total PAC-QOL score significantly decreased from 1.83 ± 0.67 to 1.17 ± 0.56 (*p* < 0.001). The number of SBMs per week increased significantly from 2.6 ± 1.2 to 4.1 ± 2.1 (*p* < 0.001). BSFS scores at baseline were “1” for 13 persons, “2” for 10 persons, “3” for 2 persons, and “4” for 2 persons. BSFS scores at 4 weeks changed to “2” for 3 persons, “3” for 5 persons, “4” for 18 persons, and “5” for 1 person. The BSFS score significantly improved from 1.9 ± 0.8 to 3.6 ± 0.7 (*p* < 0.001). Bayesian statistics confirmed the results’ significance.

**Table 3:** PAC-QOL, SBM, and Bayes factor at baseline and week 4

	Baseline Mean ± SD, median [IQR]	Week 4 Mean ± SD, median [IQR]	*P* value	Bayes factor
PAC-QOL				
Global score	1.83 ± 0.67	1.17 ± 0.56	< 0.001	< 0.001
	1.93 [1.29, 2.36]	1.14 [0.71, 1.57]		
Physical discomfort	1.94 ± 0.79	0.97 ± 0.72	< 0.001	< 0.001
	2.00 [1.25, 2.50]	1.00 [0.50, 1.50]		
Psychosocial discomfort	1.16 ± 0.93	0.63 ± 0.58	< 0.001	0.010
	0.88 [0.25, 2.00]	0.50 [0.00, 1.00]		
Worries and concerns	1.84 ± 0.73	1.27 ± 0.59	< 0.001	0.001
	2.00 [1.09, 2.45]	1.18 [0.82, 1.91]		
Satisfaction	2.79 ± 0.61	1.98 ± 0.77	< 0.001	0.002
	2.80 [2.40, 3.40]	2.00 [1.60, 2.60]		
Weekly SBM	2.6 ± 1.2	4.1 ± 2.1	< 0.001	< 0.001
	2 [2, 3]	4 [2, 5]		
Bristol Stool Form Scale score	1.9 ± 0.8	3.6 ± 0.7	< 0.001	0.007
	2 [1, 2]	4 [3, 4]		

*PAC-QOL* Patients Assessment of Constipation-Quality of Life, *SBM* spontaneous bowel movement

**Fig. 2:**
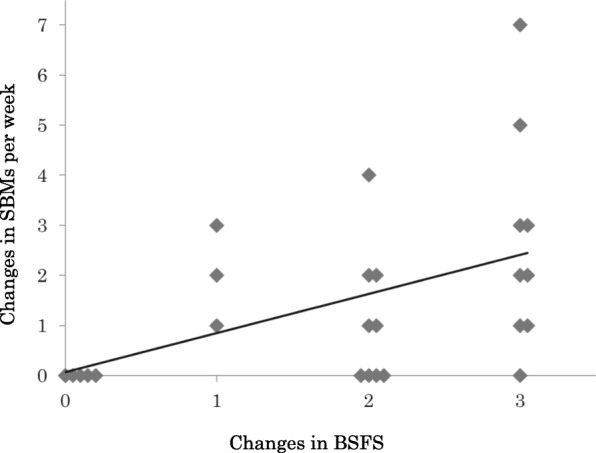
Changes in BSFS and changes in SBMs after 4-week elobixibat intake. Kendall rank correlation coefficient was 0.650 (*P* < 0.001). SBMs, spontaneous bowel movements; BSFS, Bristol Stool Form Scale

Figure 2 shows the relationship between changes in BSFS and SBMs in 4-week elobixibat intake. BSFS and SBMs were significantly related (Kendall rank correlation coefficient tau = 0.468, *p* = 0.004). Bayesian statistics moderately supported the significance of the result.

We divided the patients into two groups according to the number of laxatives.

Tables 4 and 5 show the PAC-QOL scores, SBMs, and BSFS scores at baseline and after 4 weeks in the two groups.

**Table 4:** PAC-QOL, SBM, and Bayes factor at baseline and at week 4 in patients taking 1 laxative (*N* = 13)

	Baseline Mean ± SD, median [IQR]	Week 4 Mean ± SD, median [IQR]	*P* value	Bayes factor
PAC-QOL				
Global score	1.68 ± 0.63	1.13 ± 0.42	0.002	0.007
	1.64 [1.14, 2.31]	1.00 [0.77, 1.34]		
Physical discomfort	1.79 ± 0.66	0.92 ± 0.53	0.004	0.026
	1.75 [1.00, 2.38]	0.75 [0.50, 1.13]		
Psychosocial discomfort	0.94 ± 0.88	0.48 ± 0.47	0.017	0.285
	0.75 [0.19, 1.57]	0.25 [0.07, 0.88]		
Worries and concerns	1.73 ± 0.74	1.24 ± 0.52	0.023	0.219
	2.00 [1.05, 2.32]	1.09 [0.82, 1.64]		
Satisfaction	2.66 ± 0.54	2.09 ± 0.54	0.009	0.085
	2.40 [2.30, 3.10]	1.80 [1.60, 2.50]		
Weekly SBM	2.6 ± 1.2	4.1 ± 2.1	0.005	0.071
	2 [2, 3]	4 [2, 5]		
Bristol Stool Form Scale score	1.9 ± 0.9	3.6 ± 0.6	0.004	0.010
	2 [1, 3]	4 [3, 4]

**Table 5:** PAC-QOL, SBM, and Bayes factor at baseline and week 4 in patients taking 2 or 3 laxatives (*N* = 14)

	Baseline Mean ± SD, median [IQR]	Week 4 Mean ± SD, median [IQR]	*P* value	Bayes factor
PAC-QOL				
Global score	1.97 ± 0.68	1.21 ± 0.67	0.002	0.009
	2.00 [1.44, 2.52]	1.41 [0.43, 1.69]		
Physical discomfort	2.07 ± 0.88	0.76 ± 0.64	0.002	0.020
	2.00 [1.25, 2.75]	0.82 [0.00, 1.50]		
Psychosocial discomfort	1.36 ± 0.93	0.63 ± 0.58	0.012	0.174
	1.57 [0.56, 2.13]	0.50 [0.00, 1.00]		
Worries and concerns	1.95 ± 0.71	1.30 ± 0.65	0.002	0.010
	2.09 [1.29, 2.57]	1.45 [0.64, 1.96]		
Satisfaction	2.90 ± 0.64	1.87 ± 0.93	0.004	0.056
	3.00 [2.35, 3.45]	2.00 [1.10, 2.65]		
Weekly SBM	2.3 ± 1.0	3.2 ± 1.5	0.027	0.363
	2 [2, 3]	3 [2, 4]		
Bristol Stool Form Scale score	1.8 ± 0.8	3.6 ± 0.8	0.002	0.001
	2 [1, 2]	4 [3, 4]		

As shown in Table 4, scores decreased for physical discomfort from 1.79 ± 0.66 to 0.92 ± 0.53 (*p* = 0.004); psychosocial discomfort from 0.94 ± 0.88 to 0.48 ± 0.47 (*p* = 0.017); worries/ concerns from 1.73 ± 0.74 to 1.24 ± 0.52 (*p* = 0.023); and satisfaction from 2.66 ± 0.54 to 2.09 ± 0.54 (*p* = 0.009). The total PAC-QOL score significantly decreased from 1.68 ± 0.63 to 1.13 ± 0.42 (*p* = 0.002), whereas the number of SBMs per week increased from 2.6 ± 1.2 to 4.1 ± 2.1 (*p* = 0.005). The BSFS score significantly improved from 1.9 ± 0.0 to 3.6 ± 0.6 (*p* = 0.004). Bayesian statistics confirmed the significance of these results.

As shown in Table 5, scores decreased for physical discomfort from 2.07 ± 0.88 to 0.76 ± 0.64 (*p* = 0.002); psychosocial discomfort from 1.36 ± 0.93 to 0.63 ± 0.58 (*p* = 0.012); worries/ concerns from 1.95 ± 0.71 to 1.30 ± 0.65 (*p* = 0.002); and satisfaction from 2.90 ± 0.64 to 1.87 ± 0.93 (*p* = 0.004). The total PAC-QOL score significantly decreased from 1.97 ± 0.68 to 1.21 ± 0.67 (*p* = 0.002), whereas the number of SBMs per week increased from 2.3 ± 1.0 to 3.2 ± 1.5 (*p* = 0.027). The BSFS score significantly improved from 1.8 ± 0.8 to 3.6 ± 0.8 (*p* = 0.002). Bayesian statistics confirmed the significance of these results.

Table 6 shows the levels of electrolytes and cholesterol at baseline and after 4 weeks of elobixibat 10 mg/day intake. Sodium, potassium, total calcium, phosphate, LDL, and HDL did not change significantly after elobixibat intake. Bayesian statistics confirmed the results.

**Table 6:** Electrolytes and cholesterol levels at baseline and week 4

	Baseline Mean ± SD, median [IQR]	Week 4 Mean ± SD, median [IQR]	*P* value	Bayes factor
Sodium (mEq/L)	139 ± 3139 [136, 141]	139 ± 3139 [137, 141]	0.393	4.112
Potassium (mEq/L)	4.6 ± 0.8, 4.6 [4.2, 5.1]	4.7 ± 0.8 4.7 [4.0, 5.0]	0.909	6.485
Total calcium (mg/dL)	8.5 ± 0.6, 8.5 [8.1, 8.7]	8.4 ± 0.7 8.5 [7.9, 8.8]	0.287	5.125
Phosphate (mg/dL)	4.9 ± 1.2, 4.5 [4.0, 5.8]	5.2 ± 1.3, 5.2 [4.1, 6.1]	0.119	3.973
LDL (mg/dL)	80 ± 28, 79 [54, 106]	78 ± 25, 75 [55, 107]	0.103	4.692
HDL (mg/dL)	41 ± 14, 41 [31, 50]	43 ± 14, 41 [33, 47]	0.306	3.968

*LDL* low-density lipoprotein cholesterol, *HDL* high-density lipoprotein cholesterol

## Discussion

The present study revealed that supplemental administration of elobixibat to hemodialysis patients with chronic constipation improved their PAC-QOL, SBMs, and BSFS, without significantly affecting the levels of sodium, potassium, total calcium, phosphate, LDL, and HDL. Hard stools were significantly related to SBMs, and softening stools were significantly related to increase in SBMs.

Defecation is a necessary physiological function, and its failure poses a major problem in daily life. The prevalence of chronic constipation had been about 16% in the general population [17] and was reported to be higher, at about 36 to 66%, in hemodialysis patients [2]. However, the reliability on self-reporting is debatable when diagnosing constipation because patient’s perception may not accurately indicate the actual problem.

Constipation that does not improve and unpredictable defecation can cause gradual anxiety and dissatisfaction, thereby restricting daily life activities. Indeed, chronic constipation can greatly impair a patient’s QOL, and addressing it was shown to improve both physical and mental QOL [1]. We evaluated the QOL of chronic constipation using PAC-QOL. PAC-QOL is the most widely used disease-specific quality of life measure for chronic constipation. In a recent study, PAC-QOL was also used to evaluate various clinical presentations of functional constipation, irritable bowel syndrome with constipation, and No Rome Constipation in Italy [18].

Treatment for chronic constipation usually begins with diet and lifestyle improvement as well as appropriate exercise. If these changes do not help, medications or surgery may be recommended [19]. Chronic constipation may also be associated with colonic or rectal anatomo-functional alternations as colonic inertia or rectal outlet obstruction, respectively. The re-educative treatment and rehabilitation of the harmony of the “imaginary cuboid” constituted by the diaphragm, abdominal wall, spine and pelvic floor may be important [20–22].

Elobixbat is a pure enantiomer of synthetically modified 1,5-benzothiazepine with a seven-membered heterocyclic ring attached to a benzene ring (chemical formula C_36_H_45_N_3_O_7_S_2_) [23, 24]. The main route of elimination of elobixibat is in the feces. There is little excretion in the urine. The estimated half-life in humans is less than 4 h. The binding rate to human plasma proteins in vitro is more than 99%, and the human blood cell migration rate is less than 5%. After oral ingestion of ^14^C-elobixibat, there was no accumulation of elobixibat or associated metabolites within the plasma or urine [23]. Multiple clinical trials evaluating the use of elobixibat have demonstrated consistent improvements in stool frequency and consistency and clinically meaningful end points across various populations diagnosed with chronic idiopathic constipation [5, 6, 25–27]. However, no studies have evaluated the use of this drug in dialysis patients with chronic constipation. In the current study, we demonstrated for the first time that adding elobixibat to drugs for constipation improved hemodialysis patients’ QOL.

Hemodialysis patients usually undergo dialysis three times per week. If a patient feels the urge to defecate during dialysis, the dialysis needs to be temporarily stopped. If patients cannot make it to a toilet in time, they may inadvertently excrete in the presence of medical staff and other patients in the room, which can cause great mental distress for patients. Therefore, defecation on four non-dialysis days a week, and avoiding defecation on dialysis days three times per week may be desirable. In the current study, elobixibat was shown to increase the number of SBMs from 2.6 to 4.1 times per week. Hemodialysis patients are generally considered to pass hard stools because of the restrictions in the intake of dietary fiber and water, as well as water removal by dialysis. Aging-associated muscle atrophy affects both skeletal and smooth muscles, including those in the digestive tract, thereby affecting digestive tract motor function [28]. In elderly dialysis patients, hard stools make defecation more difficult. Elobixibat softens the stool by inhibiting the reabsorption of bile acids. In this study, elobixibat significantly improved the BSFS score and changes in BSFS were related to changes in SBMs. This implied that the softened stools made defecation easier and increased weekly defecation frequency, which may have led to the improvement in the PAC-QOL.

Elobixibat was reported to lower serum LDL cholesterol by approximately 10% [29]. However, in this study, the LDL and HDL levels did not change significantly after elobixibat administration for 4 weeks. Although lubiprostone was reported to decrease serum IP levels in hemodialysis patients [30], electrolytes did not change significantly in this study.

The present study had several limitations. First, this study was conducted using questionnaires, and we could not exclude the potential effects of other unknown confounders. Second, it was retrospective observational study. Nevertheless, this was, to the best of our knowledge, the first multicenter observational study that evaluated the efficacy of elobixibat in hemodialysis patients with chronic constipation. Furthermore, this study’s reliability was confirmed by Bayesian inference. However, further investigation on a larger sample size from multiple centers is necessary for external validity. Finally, the generalizability of our results on the efficacy of elobixibat in hemodialysis patients needs to be verified in a large-scale, randomized controlled study with other drugs or without elobixibat.

## Conclusion

In conclusion, additional intake of elobixibat improved the PAC-QOL score, BSFS score, and SBM frequency in hemodialysis patients with chronic constipation, especially those with hard stools and poor stool rhythm. Further investigation is necessary to ascertain our findings.

## Data Availability

The datasets generated and/or analyzed during the current study are not publicly available to maintain patient confidentiality in a small cohort, but are available from the corresponding author on reasonable request.

## Abbreviations

ADPKD: Autosomal dominant polycystic kidney disease
BSFS: Bristol Stool Form Scale
CGN: Chronic glomerulonephritis
HDL: High-density lipoprotein cholesterol
IP: Inorganic Phosphorus
LDL: Low-density lipoprotein cholesterol
PAC-QOL: Patient Assessment of Constipation-Quality of Life
QOL: Quality of Life
SBMs: Spontaneous bowel movements

## References

[1] Wald A, Scarpignato C, Kamm MA, Mueller-Lissner S, Helfrich I, Schuijt C (2007). The burden of constipation on quality of life: results of a multinational survey. Aliment Pharmacol Ther.

[2] Zuvela J, Trimingham C, Le Leu R, Faull R, Clayton P, Jesudason S (2018). Gastrointestinal symptoms in patients receiving dialysis: a systematic review. Nephrology (Carlton).

[3] Gillberg PG, Dahlström M, Starke I, Östlund-Lindqvist A-M (2010). The IBAT inhibition by A3309–a potential mechanism for the treatment of constipation. Gastroenterology.

[4] Acosta A, Camilleri M (2014). Elobixibat and its potential role in chronic idiopathic constipation. Ther Adv Gastroenterol.

[5] Nakajima A, Seki M, Taniguchi S (2018). Determining an optimal clinical dose of elobixibat, a novel inhibitor of the ileal bile acid transporter, in Japanese patients with chronic constipation: a phase II, multicenter, double-blind, placebo-controlled randomized clinical trial. J Gastroenterol.

[6] Nakajima A, Seki M, Taniguchi S, Ohta A, Gillberg PG, Mattsson JP (2018). Safety and efficacy of elobixibat for chronic constipation: results from a randomised, double-blind, placebo-controlled, phase 3 trial and an open-label, single-arm, phase 3 trial. Lancet Gastroenterol Hepatol.

[7] Yamamoto H, Nishi S, Tomo T, Masakane I, Saito K, Nangaku M (2017). 2015 Japanese Society for Dialysis Therapy: guidelines for renal anemia in chronic kidney disease. Renal Replace Ther.

[8] Watanabe Y, Kawanishi H, Suzuki K, Nakai S, Tsuchida K, Tabei K (2015). Japanese society for dialysis therapy clinical guideline for ªmaintenance hemodialysis: hemodialysis prescriptions. Ther Apher Dial.

[9] Nakao T, Inaba M, Abe M, Kaizu K, Shima K, Babazono T (2015). Best practice for diabetic patients on hemodialysis 2012. Ther Apher Dial.

[10] Fukagawa M, Yokoyama K, Koiwa F, Taniguchi M, Shoji T, Kazama J (2013). Clinical practice guideline for the management of chronic kidney disease-mineral and bone disorder. Ther Apher Dial.

[11] Hirakata H, Nitta K, Inaba M, Shoji T, Fujii H, Kobayashi S (2012). Japanese Society for Dialysis Therapy guidelines for management of cardiovascular diseases in patients on chronic hemodialysis. Ther Apher Dial.

[12] Marquis P, De La Loge C, Dubois D, McDermott A, Chassany O (2005). Development and validation of the patient assessment of constipation quality of life questionnaire. Scand J Gastroenterol.

[13] Nomura H, Agatsuma T, Mimura T (2014). Validity and reliability of the Japanese version of the patient assessment of constipation quality of life questionnaire. J Gastroenterol.

[14] Heaton KW, Ghosh S, Braddon FE (1991). How bad are the symptoms and bowel dysfunction of patients with the irritable bowel syndrome? A prospective, controlled study with emphasis on stool form. Gut.

[15] Wasserstein RL, Lazar NA (2016). Editorial: the ASA's statement on p-values: context, process, and purpose. Am Stat.

[16] Benjamin DJ, Berger JO, Johannesson M, Nosek BA, Wagenmakers EJ, Berk R (2018). Redefine statistical significance. Nat Hum Behav.

[17] Mugie SM, Benninga MA, Di Lorenzo C (2011). Epidemiology of constipation in children and adults: a systematic review. Best Pract Res Clin Gastroenterol.

[18] Bellini M, Usai-Satta P, Bove A, Bocchini R, Galeazzi F, Battaglia E (2017). Chronic constipation diagnosis and treatment evaluation: the “CHRO.CO.DI.T.E.” study. BMC Gastroenterol.

[19] Bove A, Bellini M, Battaglia E, Bocchini R, Gambaccini D, Bove V, Pucciani F, Altomare DF, Dodi G, Sciaudone G, Falletto E, Piloni V (2012). Consensus statement AIGO/SICCR diagnosis and treatment of chronic constipation and obstructed defecation (part II: treatment). World J Gastroenterol.

[20] Brusciano L, Limongelli P, del Genio G, Sansone S, Rossetti G, Maffettone V, Napolitano V, Sagnelli C, Amoroso A, Russo G, Pizza F, del Genio A (2007). Useful parameters helping proctologists to identify patients with defaecatory disorders that may be treated with pelvic floor rehabilitation. Tech Coloproctol.

[21] Brusciano L, Limongelli P, del Genio G, Rossetti G, Sansone S, Healey A, Maffettone V, Napolitano V, Pizza F, Tolone S, del Genio A (2009). Clinical and instrumental parameters in patients with constipation and incontinence: their potential implications in the functional aspects of these disorders. Int J Color Dis.

[22] Brusciano L, Gambardella C, Tolone S, Del Genio G, Terracciano G, Gualtieri G (2019). Schiano di Visconte M, Docimo L. an imaginary cuboid: chest, abdomen, vertebral column and perineum, different parts of the same whole in the harmonic functioning of the pelvic floor. Tech Coloproctol.

[23] Chedid V, Vijayvargiya P, Camilleri M (2018). Elobixibat for the treatment of constipation. Expert Rev Gastroenterol Hepatol.

[24] Miner PB (2018). Elobixibat, the first-in-class Ileal bile acid transporter inhibitor, for the treatment of chronic idiopathic constipation. Expert Opin Pharmacother.

[25] Simren M, Bajor A, Gillberg PG, Rudling M, Abrahamsson H (2011). Randomised clinical trial: the ileal bile acid transporter inhibitor a3309 vs. placebo in patients with chronic idiopathic constipation–a double-blind study. Aliment Pharmacol Therapeut.

[26] Wong BS, Camilleri M, McKinzie S, Burton D, Graffner H, Zinsmeister AR (2011). Effects of a3309, an ileal bile acid transporter inhibitor, on colonic transit and symptoms in females with functional constipation. Am J Gastroenterol.

[27] Chey WD, Camilleri M, Chang L, Rikner L, Graffner H (2011). A randomized placebo-controlled phase iib trial of a3309, a bile acid transporter inhibitor, for chronic idiopathic constipation. Am J Gastroenterol.

[28] Gutzwiller JP, Aschwanden J, Iff S, Leuenberger M, Perrig M, Stanga Z (2011). Glucocorticoid treatment, immobility, and constipation are associated with nutritional risk. Eur J Nutr.

[29] Kumagai Y, Amano H, Sasaki Y, Nakagawa C, Maeda M, Oikawa I (2018). Effect of single and multiple doses of elobixibat, an ileal bile acid transporter inhibitor, on chronic constipation: a randomized controlled trial. Br J Clin Pharmacol.

[30] Gen S, Nobe K, Ikeda N (2016). Lubiprostone, a novel laxative, might improve hyperphosphatemia without water dilution. Renal Replace Ther.

